# Within- and Between-Child Variation in Repeated Urinary Pesticide Metabolite Measurements over a 1-Year Period

**DOI:** 10.1289/ehp.1306737

**Published:** 2013-12-10

**Authors:** Kathleen R. Attfield, Michael D. Hughes, John D. Spengler, Chensheng Lu

**Affiliations:** 1 Department of Environmental Health, and; 2 Department of Biostatistics, Harvard School of Public Health, Boston, Massachusetts, USA

## Abstract

Background: Children are exposed to pesticides from many sources and routes, including dietary and incidental ingestion, dermal absorption, and inhalation. Linking health outcomes to these exposures using urinary metabolites requires understanding temporal variability within subjects to avoid exposure misclassification.

Objectives: We characterized the within- and between-child variability of urinary organophosphorus and pyrethroid metabolites in 23 participants of the Children’s Pesticide Exposure Study–Washington over 1 year and examined the ability of one to four spot urine samples to categorize mean exposures.

Methods: Each child provided urine samples twice daily over 7- to 16-day sessions in four seasons in 2003 and 2004. Samples were analyzed for five pyrethroid and five organophosphorus (OP) metabolites. After adjusting for specific gravity, we used a customized maximum likelihood estimation linear mixed-effects model that accounted for values below the limit of detection to calculate intraclass correlation coefficients (ICC) and conducted surrogate category analyses.

Results: Within-child variability was 2–11 times greater than between-child variability. When restricted to samples collected during a single season, ICCs were higher in the fall, winter, and spring than in summer for OPs, and higher in summer and winter for pyrethroids, indicating an increase in between-person variability relative to within-person variability during these seasons. Surrogate category analyses demonstrated that a single spot urine sample did not categorize metabolite concentrations well, and that four or more samples would be needed to categorize children into quartiles consistently.

Conclusions: Urinary biomarkers of these short half-life pesticides exhibited substantial within-person variability in children observed over four seasons. Researchers investigating pesticides and health outcomes in children may need repeated biomarker measurements to derive accurate estimates of exposure and relative risks.

Citation: Attfield KR, Hughes MD, Spengler JD, Lu C. 2014. Within- and between-child variation in repeated urinary pesticide metabolite measurements over a 1-year period. Environ Health Perspect 122:201–206; http://dx.doi.org/10.1289/ehp.1306737

## Introduction

Insect control in U.S. agriculture and residences is currently predominated by the widespread use of pesticides, amounting to > 90 million pounds annually (Grube et al. 2011). This results in dietary exposures from the residues left behind from organophosphorus (OP) and pyrethroid insecticides used on food and animal feed crops [U.S. Environmental Protection Agency (EPA) 2006a]. After the 2001 U.S. EPA voluntary phaseout of residential OP use, pyrethroids have become the principal pesticide class used for indoor pest control (Sudakin 2006), creating opportunities for dermal, inhalation, and incidental ingestion exposures (Agency for Toxic Substances and Disease Registry 2003). In addition to acute poisonings, epidemiological studies have reported evidence of neurotoxic and developmental effects from OP exposures (Bouchard et al. 2010; Harley et al. 2011; Rauh et al. 2011) and evidence of reproductive toxicity (Meeker et al. 2008; Nassr et al. 2010) and endocrine disruption (Han et al. 2008; Meeker et al. 2009) associated with pyrethroid exposures. In addition, the U.S. EPA has listed permethrin, a widely used pyrethroid for both agricultural and residential insect control, as “likely to be carcinogenic to humans” (U.S. EPA 2006b).

To investigate these potential risks, urinary pesticide metabolites are often used as biomarkers of exposure. Integrating exposures across sources and routes, biomarkers are more likely to give a better indication of actual absorption at the individual level than environmental measures. Urine biomarkers often are examined because they are easier and less invasive to collect than blood or tissue samples. Although all biomarkers are influenced by the timing, magnitude, and frequency of exposure as well as biological clearance rates, for short half-life chemicals with irregular exposure patterns, such as the pyrethroid and OP pesticides, urinary metabolite levels may be especially variable not just between people but even within a person.

When using biomarkers to represent an individual’s exposure to these short half-life chemicals, the within-person variability, if not properly accounted for, could lead to exposure measurement error or misclassification and obscure results of epidemiologic investigations and assessments of risk. But researchers have relied on the postulation that the between-person variation in pesticide metabolites, because of both different exposure patterns and metabolic processing of these pesticides, will predominate and differentiate their participants (Adgate et al. 2001; Egeghy et al. 2011). Investigations of within-person variability are necessary to understand the diversity of exposure and to design effective studies for understanding pesticide effects upon health.

Information on within-person diversity of exposures to OPs and pyrethroids is mounting. In a study of 11 adult males, Meeker et al. (2005) reported that within-person variability in urine concentrations of the OP metabolite 3,5,6-trichloro-2-pyridinol (TCPy) was five times higher than between-person variability with multiple measurements taken over 3 consecutive months. Furthermore, MacIntosh et al. (1999) reported that the average within-person range of TCPy concentrations in 80 adults with an average of 4.3 measurements over 1 year was 1.5 times higher than the median TCPy concentrations for the study population as a whole.

Estimates of within-person variability based on adult studies, however, may not be valid for children because of differences in exposure patterns (e.g., greater hand-to-mouth contact, time close to the ground, food and liquid ingestion, likelihood of non-food ingestion, and air inhalation, relative to their body size compared with adults) (Roberts et al. 2009) and differences in biological processing (e.g., higher metabolic rates and immature detoxification processes) (Morgan et al. 2005; Roberts et al. 2009). Potential health effects of pesticide exposures are of particular concern in children because their exposures are greater relative to their body weight, and because their organ systems are still developing (Landrigan et al. 1999). Among studies of short duration (up to 7 days), two reported that between-child variability was dominant although within-child variability was still substantial (Adgate et al. 2001; Egeghy et al. 2011); in a third study population, within-child variability was higher than between-child variability (Bradman et al. 2013). In a longer-term study of an agriculturally exposed population followed over 21 months, within-child variability accounted for > 90% of the total variability for nonspecific OP metabolites (Griffith et al. 2011). To our knowledge, only one study has examined the long-term variability of OP exposures in children from the general population (Sexton and Ryan 2012). In that population, there was a nonsignificant predominance of within-child variability for OP metabolites measured in four samples collected over 2 years. Additionally, no study has evaluated the variability of pyrethroid exposures over the long term. More information is needed to understand the extent of variability within the general population.

In the present study, we used numerous repeated-measures data from a study of children’s dietary pesticide exposures in Washington state (Lu et al. 2008, 2009) to investigate the extent to which within-child variability contributed to the overall variability in metabolites of OP and pyrethroid pesticides over four seasons of sampling. Second, to shed light on how many samples might be needed in a situation of high within-person variability, we conducted an analysis to assess participant assignment into exposure quartiles according to the number of measurements used.

## Methods

*Study design*. This study is part of the Children’s Pesticide Exposure Study–Washington (CPES-WA) in which 23 children, 3–11 years of age and living in suburban Seattle, Washington, participated in an organic diet substitution study from 2003 to 2004; details have been reported elsewhere (Lu et al. 2008, 2009). Briefly, the children were recruited from two local public elementary schools and one Montessori preschool. The children participated in consecutive day urine sampling periods in July/August 2003 (median, 15 days; range, 15–16 days); October/November 2003 (median, 12 days; range, 11–13 days); January/February 2004 (median, 7 days; range, 7–8 days); and April/May 2004 (median, 7 days; range, 5–9 days). In the summer and fall sampling periods, an organic diet substitution phase (from day 4 to day 8) was incorporated into the study design to assess the dietary pesticide exposures. For the present study we included only samples collected during the conventional diet portions of the study so that metabolites measured in the urine samples would be representative of typical exposures in the children. Participating children in each session numbered 23 in the summer, 21 in the fall, 20 in the winter, and 19 in the spring. Written informed consent was provided by older children and by the parents of all participants, and oral assent was provided by younger children. The study was approved by the University of Washington Human Subjects Division.

*Urine collection and laboratory analysis*. Each child provided two urine samples per day: the last void before bedtime and the following first morning void. Previous studies have demonstrated that first voids are good predictors of overall daily exposure for OPs (Kissel et al. 2005), and in combination with last voids, a large portion of the daily exposure is represented. Additional spot urine samples collected at different times of the day during the study were excluded from the present analysis to increase the comparability of the samples evaluated.

After collection, urine samples were stored on ice or refrigerated before processing in the laboratory and then stored at –20^o^C. Samples were analyzed at the National Center for Environmental Health at the Centers for Disease Control and Prevention (CDC; Atlanta, GA) using high performance liquid chromatography–tandem mass spectrometry (Olsson et al. 2004). Target OP metabolites were malathion dicarboxylic acid (MDA), TCPy, 2-isopropyl-4-methyl-6-hydroxypyrimidinol (IMPy), and 2-diethylamino-6-methyl-pyrmidin-4-ol (DEAMPy). Target pyrethroid insecticide metabolites were 3-phenoxybenzoic acid (PBA), 4-fluoro-3-phenoxybenzoic acid (4F3PBA), *cis*-2,2-(dichloro)-2-dimethylvinylcyclopropane carboxylic acid (*cis*-DCCA), *trans*-2,2-(dichloro)-2-dimethylvinylcyclopropane carboxylic acid (*trans*-DCCA), and *cis*-2,2-(dibromo)-2-dimethylvinyl-cyclopropane carboxylic acid (DBCA).

*Data analysis*. Metabolite concentrations were adjusted for specific gravity to control for dilution using a reference specific gravity of 1.019 g/cm^3^, the 2007–2008 National Health and Nutrition Examination Survey (NHANES) mean for children 6–11 years of age (CDC 2009; Levine and Fahy 1945). Because carryover from a previous day could be expected due to the biological half-life (hours to a couple of days) of these compounds, we confirmed that the conventional diet days following the end of the organic diet portions of the original study were not significantly different from other conventional diet days before including them in the analyses (*t*-test *p*-value > 0.05).

Intraclass correlation coefficients (ICC), defined as the ratio of between-subject variance to total variance, were calculated as a measure of the reproducibility of measurements over time within individuals. ICCs can range from 0 to 1; ≥ 0.75 indicates excellent reproducibility and ≤ 0.4 indicates poor reproducibility (Rosner 2006). Between- and within-subject variances were calculated with a linear mixed effects model using maximum likelihood estimation (MLE) modified to account for values below the limit of detection (LOD) and repeated measurements as implemented in SAS 9.3 (SAS Institute Inc., Cary, NC) using PROC NLMIXED, assuming a compound symmetry covariance structure (Jin et al. 2011). Age (3–6, 7–11 years), sex, and season were included as covariates in the model:

*Ln*(*Y*) = β_0_ + β_1_(age) + β_2_(sex) + β_3_(season) + *b*_1_ + ε, [1]

where *Y* is the metabolite concentration adjusted for specific gravity, *b*_1_ is the between-subject random effect, and ε is the within-subject error.

For metabolites with high percentages of nondetects, the model’s stipulation of a normal distribution of the data was difficult to evaluate. Therefore, we restricted the ICC analyses to the four metabolites (MDA, TCPy, PBA, and *trans*-DCCA) that were > LOD in > 50% of samples. Data were natural log-transformed before analysis. We performed a sensitivity analysis on the calculation of the ICCs by substituting LOD/2 for values < LOD and using an unmodified NLMIXED procedure. A further sensitivity analysis was used to test the model’s sensitivity to the assumption of equal covariance. The ICC calculations were repeated using a subset of the data with an equal covariance pattern, where only samples with at least 2 intervening days were included in the subset per season.

**Figure 1:**
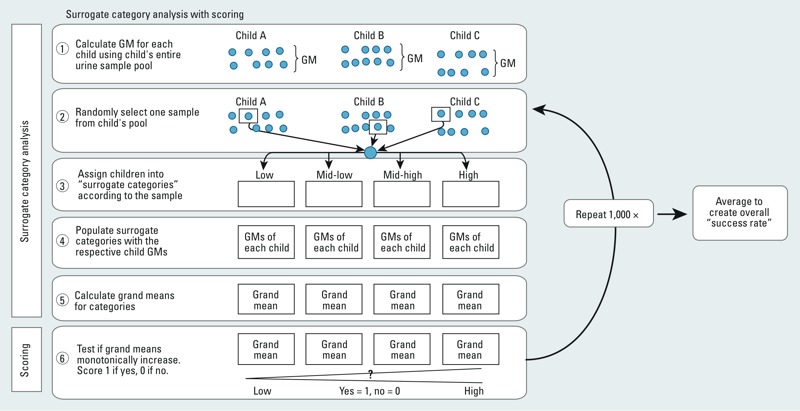
Process of surrogate category analysis with scoring. GM, geometric mean.

To address how much exposure misclassification may develop when participants are categorized into exposure groups and how many samples may be necessary to improve the categorization, we performed surrogate category analyses for the first and last voids separately (Hauser et al. 2004; Willet 1998) with an additional scoring step (Figure 1). We calculated the geometric mean value of a metabolite across all samples collected from each participant, resulting in 23 participant mean values. Next, we assigned each participant to an exposure quartile (a “surrogate category”) based on the metabolite concentration of a single sample selected at random from each participant’s pool of samples. Then we populated each surrogate category with the children’s geometric means and calculated the group grand means. Then we evaluated the performance of the category assignment. It would not be possible to directly determine whether individuals were correctly assigned according to their “true” and unobserved distribution in the population; however, if surrogate categories were correctly assigned, the mean value of each category should increase monotonically from the lowest to the highest exposure category. If this were the case, we assigned the run a score of 1, and 0 otherwise. Then we repeated the sampling and classification steps 1,000 times and used the mean value of the 1,000 scores (expressed as a percentage) to indicate an average “success rate.” We performed this process three additional times based on the mean value of two, three, and four randomly selected samples for each participant. For this analysis, we substituted instrument-read values (when available), or the LOD/2, as the concentration for all samples with measurements < LOD.

## Results

A total of 1,215 urine samples were collected from 23 children (15–63 per child, with a median of 59) during the conventional diet stages of the study over four seasons. Metabolite frequency of detection, and distributions, adjusted for specific gravity for first and last voids, are presented in Table 1. Summary statistics of unadjusted levels for both the conventional and organic diet parts of the sampling were previously reported (Lu et al. 2008, 2009). The frequency of detection varied among the metabolites. Those detected most frequently across all measurements were PBA (> 85%), a common metabolite of several pyrethroids, and TCPy, a specific metabolite of chlorpyrifos (> 81%). Several metabolites were detected < 20% of the time, including the pyrethroid metabolites 4F3PBA and DBCA (specific metabolites of cyfluthrin and deltamethrin, respectively), and the OP metabolites IMPy and DEAMPy.

**Table 1:** Descriptive statistics of first and last void concentrations of urinary metabolites adjusted for specific gravity from 23 children.

Metabolite	Void	Total no. of urine samples	LOD (μg/L)	Detection frequency (%)	Percentile				
					25th	50th	75th	95th	100th
Pyrethroid
PBA	First	616	0.1	91	0.5	1.1	1.5	4.5	61.2
	Last	599	0.1	85	0.3	1.0	1.4	4.1	26.5
4F3PBA	First	599	0.2	16	< LOD	< LOD	< LOD	1.1	2.3
	Last	599	0.2	13	< LOD	< LOD	< LOD	1.0	61.2
*cis*-DCCA	First	599	0.2	37	< LOD	< LOD	0.7	1.7	46.0
	Last	599	0.2	32	< LOD	< LOD	0.4	1.3	4.6
*trans*-DCCA	First	599	0.4	55	< LOD	0.9	1.4	4.4	97.1
	Last	599	0.4	49	< LOD	0.6	1.3	3.9	27.7
DBCA	First	599	0.1	3	< LOD	< LOD	< LOD	< LOD	0.3
	Last	599	0.1	4	< LOD	< LOD	< LOD	< LOD	0.8
OP
MDA	First	616	0.3	57	< LOD	0.8	3.0	13.8	433.8
	Last	599	0.3	52	< LOD	0.6	3.2	16.9	283.8
TCPy	First	616	0.2	87	1.0	3.8	6.8	13.0	43.8
	Last	599	0.2	81	0.8	3.2	6.0	12.2	69.5
IMPy	First	600	0.7	3	< LOD	< LOD	< LOD	< LOD	16.2
	Last	586	0.7	4	< LOD	< LOD	< LOD	< LOD	31.4
DEAMPy	First	597	0.2	10	< LOD	< LOD	< LOD	0.6	26.7
	Last	593	0.2	18	< LOD	< LOD	< LOD	1.5	50.2
Abbreviations: DBCA, *cis*-2,2-(dibromo)-2-dimethylvinyl-cyclopropane carboxylic acid; *cis*-DCCA, *cis*-2,2-(dichloro)-2-dimethylvinylcyclopropane carboxylic acid; *trans*-DCCA, *trans*-2,2-(dichloro)-2-dimethylvinylcyclopropane carboxylic acid; DEAMPy, 2-diethylamino-6-methyl-pyrmidin-4-ol; IMPy-2-isopropyl-4-methyl-6-hydroxypyrimidinol); MDA, malathion dicarboxylic acid; OP, organophosphorus; PBA, 3-phenoxybenzoic acid; 4F3PBA, 4-fluor-3-phenoxybenzoic acid; 3,5,6-trichloro-2-pyridinol; TCPy, 3,5,6-trichloro-2-pyridinol.									

Within-subject variability was the larger component of variance across all analytes, making up > 65% of the total variability in first voids and last voids (Table 2). The ICCs ranged from 0.29 to 0.35 among the pyrethroid metabolites and 0.08 to 0.12 for the OP metabolites, demonstrating that within-subject variability exceeded between-subject variability by a factor of 2–11.

**Table 2:** Components of variance^*a*^ and intraclass correlation coefficients for first and last voids.

Metabolites	First void				Last void			
	Percent > LOD	Variance between	Variance within	ICC	Percent > LOD	Variance between	Variance within	ICC
PBA	91	0.48	0.97	0.33	85	0.47	1.16	0.29
*trans*-DCCA	55	0.45	0.88	0.34	49	0.49	0.91	0.35
MDA	57	0.36	4.04	0.08	52	0.56	4.88	0.10
TCPy	87	0.21	1.75	0.11	81	0.29	2.09	0.12
Abbreviations: *trans*-DCCA, *trans*-2,2-(dichloro)-2-dimethylvinylcyclopropane carboxylic acid; MDA, malathion dicarboxylic acid; OP, organophosphorus; PBA, 3-phenoxybenzoic acid; TCPy, 3,5,6-trichloro-2-pyridinol. ^***a***^Age, sex, and season were included as covariates. Metabolite concentrations were ln-transformed.								

**Figure 2:**
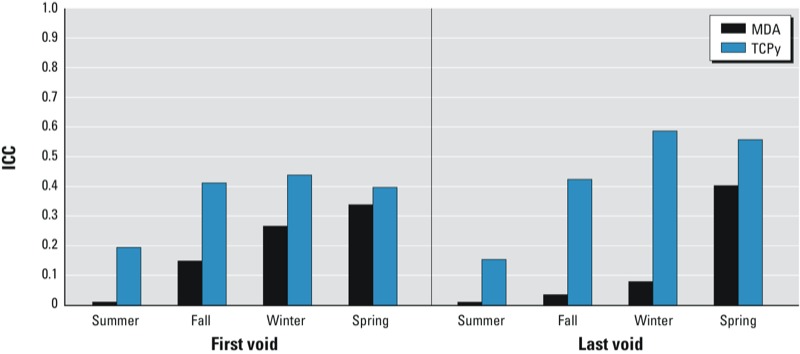
OP metabolite intraclass correlation coefficients of first and last voids by season.

**Figure 3:**
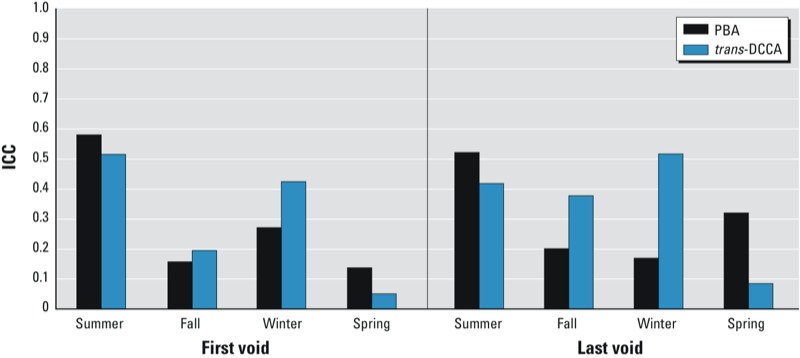
Pyrethroid metabolite intraclass correlation coefficients of first and last voids by season.

A seasonal effect on the ratio of within- and between-subject variability was observed when the same model was fit separately for each season. Compared with the overall results, ICCs increased in the fall, winter, and spring, especially for the last voids of TCPy (up to 0.59) (Figure 2). However, none reached a level considered to be a highly reproducible measure (> 0.75). For the pyrethroids (Figure 3), ICCs continued to have a large contribution of within-subject variation, although summer and winter ICCs reached or exceeded 0.5. Where ICCs increased, the within-subject variance had decreased while the between-subject variance had increased relative to the full model.

The surrogate category analysis indicated that when a single sample was used to categorize exposure into quartiles, the quartile mean values increased monotonically only 14–51% of the time (Table 3). Single samples were least likely to produce monotonically increasing quartiles for the OP metabolite MDA, and most likely to do so for the pyrethroid metabolite PBA. As the number of samples increased, the rate of successful ranking improved, to the point that PBA reached a 78% success rate with four first morning samples, and an 86% success rate for four evening samples.

**Table 3:** Surrogate category analysis based on 1–4 random samples in 1,000 resamples.

Metabolites	Void	1 Sample success rate (%)	2 Sample^*a*^ success rate (%)	3 Sample^*a*^ success rate (%)	4 Sample^*a*^ success rate (%)
PBA	First	47	50	73	78
	Last	51	61	79	86
*trans*-DCCA	First	48	47	67	71
	Last	48	50	66	78
MDA	First	15	31	52	59
	Last	14	32	55	66
TCPy	First	19	34	44	55
	Last	32	41	59	66
Abbreviations: *trans*-DCCA, *trans*-2,2-(dichloro)-2-dimethylvinylcyclopropane carboxylic acid; MDA, malathion dicarboxylic acid; PBA, 3-phenoxybenzoic acid; TCPy, 3,5,6-trichloro-2-pyridinol. Results indicate the success rate of all resamples that produced monotonically increasing quartiles. ^***a***^In runs with two or more random samples, children were assigned to quartiles according to the mean of the logged values of the selected samples.					

## Discussion

Our analyses demonstrated that most of the variance in repeated measures of OP and pyrethroid metabolites was attributable to within-subject variability in both the first and last voids of the day, with some variation in the extent of within-person variability according to season. The low ICCs we observed indicated poor reproducibility of a single measurement and the need for repeated sampling to characterize individuals’ exposures appropriately.

Seasonal variability in ICCs may offer insight into exposure sources. For the OPs, ICCs were larger in fall through spring than in the summer, which may reflect seasonal variation in food sources. Diet has been shown in previous studies to be a primary contributor to urinary OP levels (Bradman et al. 2011; Lu et al. 2009; Morgan et al. 2011), and imported fruits and vegetables have been found to have higher levels of OP residues than domestic produce (U.S. EPA 2006a). ICCs for both pyrethroid metabolites were higher in the summer than in the fall or spring. This might reflect increased use of pesticides to treat pests by those who use pesticides, which would result in more consistent environmental exposures, and thus reduced within-person variability. However, this would not explain high ICCs for *trans*-DCCA during the winter.

As a result of having many repeated measures, we were able to investigate how many samples would be sufficient to create quartiles of increasing exposure levels of participants for the entire period. Although this exploration does not indicate whether participants have been assigned to the correct quartile, it does indicate whether the quartile averages follow expected stepwise increases, which is a good starting point for any epidemiological investigation. Monotonically increasing quartiles were produced only 14–32% of the time when OP exposures were categorized based on a single sample measurement, but increased to 55–66% when categorization was based on the mean value of four samples. For pyrethroids, monotonically increasing quartiles resulted about 50% of the time when based on a single measurement, but increased to 71–86% when categorization was based on the mean value of four samples. These findings suggest that having a small number of samples from each study participant may lead to a high probability of exposure misclassification by incorrect quantile assignment and offer little assurance for correctly classifying the exposure into a specific category.

The degree of within-person variability seen in the CPES-WA children was consistent with prior studies of children’s exposures to OPs, although those relied primarily on fewer samples per person for their calculations. Similar to our study, when samples were spread over time (2 weeks to 1 year), ICCs were low (0.02–0.3), indicating a strong contribution of within-person variability (Griffith et al. 2011; Harris et al. 2010; Meeker et al. 2005; Sexton and Ryan 2012; Whyatt et al. 2009). These low ICCs were observed across different exposure scenarios: occupational parental exposure, residence in proximity to agricultural fields, and urban and suburban general population level exposure. When samples were taken in closer proximity to each other, as for six samples taken within 48 hr in the U.S. EPA study of preschool children in Ohio and North Carolina, ICCs were higher (0.44–0.65) (Egeghy et al. 2011). This indicated a greater contribution of between-child variability, but still such a substantial contribution of within-child variability that authors expressed concern about the utility of using one sample to rank participants’ 48-hr exposure levels (Egeghy et al. 2011). However, a recent study of agriculturally exposed children found low ICCs for samples taken over a single week (0.27–0.35) (Bradman et al. 2013). Similar to our study, between-subject variability increased when ICCs were calculated by season in a study of turf workers sampled up to 18 times for five pesticides (Harris et al. 2010). For pyrethroids, only a single study is available for comparison though with a much shorter time frame (2 days). The Ohio U.S. EPA study authors reported an ICC of 0.69, which, again, they suggested was not high enough to merit using single spot samples to represent the 2-day sampling period (Egeghy et al. 2011).

Our study’s generalizability could be limited by the study population’s characteristics that could influence pesticide exposures patterns and their absorption and metabolism. All children in the present study originated from the Seattle area, were Caucasian, and were of a mid- to upper range of socioeconomic status in relation to average U.S. levels. Although a direct comparison to NHANES is not possible due to its cross-sectional nature, the present study’s mean values over all samples collected during the conventional diet days for PBA, *trans*-DCCA, and TCPy were similar to mean values based on single spot urine samples collected from 6- to 11-year-old NHANES participants in 1999–2000. However, MDA was rarely detected in samples from the NHANES participants, and concentrations were much lower [95th percentile values of 2 ng/mL compared with 16 ng/mL in the present study; lower percentiles were < LOD (2.64 ng/mL)]. Although participation in the diet intervention may have made the families more conscious of their pesticide use over time, pesticide use by families was indeed reported, and the means of postorganic conventional diet portions were equivalent to or higher than preorganic portions (data not shown). We were also limited by the small number of children in the study. However, our finding of low ICCs for the pesticides evaluated is consistent with previous studies, which suggests that high within-person variability may be common across populations. Our analysis lacked replicate samples (split samples measured again on the same day or a different day) to examine the contribution of the method variability to the overall variability. However, even with a worst-case scenario of a 25% coefficient of variation (CV), our method variability could at most contribute 7% of the within-person variability. Methods commonly used for these compounds report CVs for quality controls at < 10% (Olsson et al. 2004). Also, a limit of quantitation (LOQ) was not provided by the laboratory, so we used the LOD as the censoring point in our statistical analysis. However, when we employed an estimated LOQ of LOD × 3 as the censoring point, we observed ICCs very similar to those obtained using the LOD (at most increased by 0.09; data not shown). A further limitation of the method was its reliance upon an assumption of equal covariance among the repeated measures within a subject by requiring a compound symmetry covariance structure. However, a sensitivity analysis using a subset of the data conforming to compound symmetry structures (samples with at least 2 intervening days) produced similar ICCs.

The use of an MLE approach that includes the samples < LOD in the estimation of the likelihood was a strength of our ICC analysis; this allowed us to employ the entire data set without relying on the use of substitute values. This method is not widely used in exposure assessment and environmental epidemiology to incorporate values < LOD, although it can provide estimators that are consistent, asymptotically unbiased, and efficient (Jin et al. 2011). Other methods used to account for values < LOD, including substitution of censored values with the LOD/2 or LOD/_√_^–^2, model-based multiple imputation, or reverse Kaplan–Meier estimation, cannot accommodate both repeated measurements and censored values (Jin et al. 2011). Through modeling, Jin et al. (2011) demonstrated the general improved performance of a customized MLE method over substitution in estimating group means, differences in group means, and within-subject variances, especially with large percentages of values < LOD. However, in specific scenarios of low sample numbers, high geometric standard deviation, and/or high detection frequencies, more common techniques may be equivalent or preferable. In our data, when the method was repeated with LOD/2 substitution, ICCs were higher for the pyrethroids but were approximately the same for the OPs (data not shown).

The high within-child variability and low categorization success rates demonstrated here have implications for epidemiological studies and risk assessment of children’s exposure to pesticides and health effects. Although researchers need to balance the costs and administrative load of measuring an appropriate number of people an adequate number of times, substantial random within-person errors can result in attenuated coefficients of regression and correlation, as well as bias risk estimates toward the null for continuous data and in either direction for categorical data (Willet 1998). To provide an example of the effect of the within-subject variability upon a true risk estimate, we employed the formula of Hofmann et al. (2011) derived from Rosner et al. (1992) for a case–control study with matched sets: RR_obs_ = exp[ICC × ln(RR_true_)], where RR indicates relative risk. For a true odds ratio of 2, our ICCs suggest that estimated odds ratios in studies might be as low as 1.06 (using the lowest ICC of 0.08 for MDA) to 1.27 (using the highest ICC of 0.35 for *trans-*DCCA), suggesting the possibility of substantial bias that could result in false negative findings. Large studies are not immune to the effects of variability in exposure measurement. Attenuation bias reflects high within-subject variance when measuring risk factors (relative to between-subject variance) and is not reduced by increased sample size, as demonstrated in the Framingham Heart Study (Rosner et al. 1992). Future researchers may want to consider methods for estimate adjustment or sensitivity/bias analysis to address measurement error and within-person variability in their studies (Guo et al. 2012; Rosner et al. 1992; Spiegelman 2010). Our ICCs may be useful reference values in studies of children’s pesticide biomarkers where internal validation data are not available for conducting sensitivity analyses.

In addition to better characterizing average exposures, collecting repeated samples of a short half-life chemical such as these pesticides enables a better understanding of exposure patterns and identification of extreme values. Without longitudinal data, the ability to capture trends over time or over season will be lost. Repeated measures also provide more opportunities to identify risk factors for heightened exposures, and therefore provide insight on how to reduce exposures. In this population, intermittent urinary metabolite peaks were observed and then traced back to parental uses of pesticides in or around the home (Lu et al. 2009).

In conclusion, the short half-life pyrethroid and OP pesticides exhibited substantial within-subject variability as urinary biomarkers in children when observed over four seasons of measurements. Researchers investigating exposure and risk patterns in children and links to health outcomes may need repeated measurements to derive accurate findings.

## References

[r1] Adgate JL, Barr DB, Clayton CA, Eberly LE, Freeman NC, Lioy PJ (2001). Measurement of children’s exposure to pesticides: analysis of urinary metabolite levels in a probability-based sample.. Environ Health Perspect.

[r2] Agency for Toxic Substances and Disease Registry. (2003). Toxicological Profile for Pyrethrins and Pyrethroids. Atlanta, GA:Agency for Toxic Substances and Disease Registry.. http://www.atsdr.cdc.gov/toxprofiles/tp.asp?id=787&tid=153.

[r3] Bouchard MF, Bellinger DC, Wright RO, Weisskopf MG (2010). Attention-deficit/hyperactivity disorder and urinary metabolites of organophosphate pesticides.. Pediatrics.

[r4] Bradman A, Castorina R, Barr DB, Chevrier J, Harnly ME, Eisen EA (2011). Determinants of organophosphorus pesticide urinary metabolite levels in young children living in an agricultural community.. Int J Environ Res Public Health.

[r5] Bradman A Kogut K Eisen EA Jewell NP Quiros-Alcala L Castorina R 2013 Variability of organophosphorous pesticide metabolite levels in spot and 24-hr urine samples collected from young children during 1 week. Environ Health Perspect 121 118 124; 10.1289/ehp.1104808 23052012 PMC3553429

[r6] CDC (Centers for Disease Control and Prevention). (2009). Fourth National Report on Human Exposure to Environmental Chemicals. Hyattsville, MD:National Center for Health Statistics.. http://www.cdc.gov/exposurereport/pdf/fourthreport.pdf.

[r7] Egeghy PP, Cohen Hubal EA, Tulve NS, Melnyk LJ, Morgan MK, Fortmann RC (2011). Review of pesticide urinary biomarker measurements from selected US EPA children’s observational exposure studies.. Int J Environ Res Public Health.

[r8] Griffith W, Curl CL, Fenske RA, Lu CA, Vigoren EM, Faustman EM (2011). Organophosphate pesticide metabolite levels in pre-school children in an agricultural community: within- and between-child variability in a longitudinal study.. Environ Res.

[r9] Grube A, Donaldson D, Kiely T, Wu L. (2011). Pesticides Industry Sales and Usage: 2006 and 2007 Market Estimates.

[r10] Guo Y, Little RJ, McConnell DS (2012). On using summary statistics from an external calibration sample to correct for covariate measurement error.. Epidemiology.

[r11] Han Y, Xia Y, Han J, Zhou J, Wang S, Zhu P (2008). The relationship of 3-PBA pyrethroids metabolite and male reproductive hormones among non-occupational exposure males.. Chemosphere.

[r12] Harley KG Huen K Schall RA Holland NT Bradman A Barr DB 2011 Association of organophosphate pesticide exposure and paraoxonase with birth outcome in Mexican-American women. PLoS One 6 8 e23923; 10.1371/journal.pone.0023923 21904599 PMC3164135

[r13] Harris SA, Villeneuve PJ, Crawley CD, Mays JE, Yeary RA, Hurto KA (2010). National study of exposure to pesticides among professional applicators: an investigation based on urinary biomarkers.. J Agric Food Chem.

[r14] Hauser R Meeker JD Park S Silva MJ Calafat AM 2004 Temporal variability of urinary phthalate metabolite levels in men of reproductive age. Environ Health Perspect 112 1734 1740; 10.1289/ehp.7212 15579421 PMC1253667

[r15] Hofmann JN, Yu K, Bagni RK, Lan Q, Rothman N, Purdue MP (2011). Intra-individual variability over time in serum cytokine levels among participants in the prostate, lung, colorectal, and ovarian cancer screening trial.. Cytokine.

[r16] Jin Y, Hein MJ, Deddens JA, Hines CJ (2011). Analysis of lognormally distributed exposure data with repeated measures and values below the limit of detection using SAS.. Ann Occup Hyg.

[r17] Kissel JC, Curl CL, Kedan G, Lu C, Griffith W, Barr DB (2005). Comparison of organophosphorus pesticide metabolite levels in single and multiple daily urine samples collected from preschool children in Washington state.. J Expo Anal Environ Epidemiol.

[r18] Landrigan PJ, Claudio L, Markowitz SB, Berkowitz GS, Brenner BL, Romero H (1999). Pesticides and inner-city children: exposures, risks, and prevention.. Environ Health Perspect.

[r19] Levine L, Fahy J (1945). Evaluation of urinary lead concentrations. I. The significance of the specific gravity.. J Ind Hyg Toxicol.

[r20] Lu C Barr DB Pearson MA Walker LA Bravo R 2009 The attribution of urban and suburban children’s exposure to synthetic pyrethroid insecticides: a longitudinal assessment. J Expo Sci Environ Epidemiol 19 1 69 78; 10.1038/jes.2008.49 18766203

[r21] Lu C Barr DB Pearson MA Waller LA 2008 Dietary intake and its contribution to longitudinal organophosphorus pesticide exposure in urban/suburban children. Environ Health Perspect 116 537 542; 10.1289/ehp.10912 18414640 PMC2290988

[r22] MacIntosh DL, Needham LL, Hammerstrom KA, Ryan PB (1999). A longitudinal investigation of selected pesticide metabolites in urine.. J Expo Anal Environ Epidemiol.

[r23] Meeker JD, Barr DB, Hauser R (2008). Human semen quality and sperm DNA damage in relation to urinary metabolites of pyrethroid insecticides.. Hum Reprod.

[r24] Meeker JD, Barr DB, Hauser R (2009). Pyrethroid insecticide metabolites are associated with serum hormone levels in adult men.. Reprod Toxicol.

[r25] Meeker JD, Barr DB, Ryan L, Herrick RF, Bennett DH, Bravo R (2005). Temporal variability of urinary levels of nonpersistent insecticides in adult men.. J Expo Anal Environ Epidemiol.

[r26] Morgan MK, Sheldon LS, Croghan CW, Jones PA, Robertson GL, Chuang JC (2005). Exposures of preschool children to chlorpyrifos and its degradation product 3,5,6-trichloro-2-pyridinol in their everyday environments.. J Expo Anal Environ Epidemiol.

[r27] Morgan MK Sheldon LS Jones PA Croghan CW Chuang JC Wilson NK 2011 The reliability of using urinary biomarkers to estimate children’s exposures to chlorpyrifos and diazinon. J Expo Sci Environ Epidemiol 21 280 290; 10.1038/jes.2010.11 20502492

[r28] Nassr AC, Arena AC, Toledo FC, Bissacot DZ, Fernandez CD, Spinardi-Barbisan AL (2010). Effects of gestational and lactational fenvalerate exposure on immune and reproductive systems of male rats.. J Toxicol Environ Health A.

[r29] Olsson AO, Baker SE, Nguyen JV, Romanoff LC, Udunka SO, Walker RD (2004). A liquid chromatography–tandem mass spectrometry multiresidue method for quantification of specific metabolites of organophosphorus pesticides, synthetic pyrethroids, selected herbicides, and DEET in human urine.. Anal Chem.

[r30] Rauh V Arunajadai S Horton M Perera F Hoepner L Barr DB 2011 Seven-year neurodevelopmental scores and prenatal exposure to chlorpyrifos, a common agricultural pesticide. Environ Health Perspect 119 1196 1201; 10.1289/ehp.1003160 21507777 PMC3237355

[r31] Roberts JW, Wallace LA, Camann DE, Dickey P, Gilbert SG, Lewis RG (2009). Monitoring and reducing exposure of infants to pollutants in house dust.. Rev Environ Contam Toxicol.

[r32] Rosner B. (2006). Fundamentals of Biostatistics. 6th ed.

[r33] Rosner B, Spiegelman D, Willett WC (1992). Correction of logistic regression relative risk estimates and confidence intervals for random within-person measurement error.. Am J Epidemiol.

[r34] Sexton K, Ryan AD (2012). Using exposure biomarkers in children to compare between-child and within-child variance and calculate correlations among siblings for multiple environmental chemicals.. J Expo Sci Environ Epidemiol.

[r35] Spiegelman D (2010). Approaches to uncertainty in exposure assessment in environmental epidemiology.. Annu Rev Public Health.

[r36] Sudakin DL (2006). Pyrethroid insecticides: advances and challenges in biomonitoring.. Clin Toxicol.

[r37] U.S. EPA (U.S. Environmental Protection Agency). (2006a). Supplemental Report: Details on Dietary Risk Data in Support of Report No. 2206-P-0028, Measuring the Impact of The Food Quality Protection Act: Challenges and Opportunities. Washington, DC:U.S. EPA Office of Inspector General.. http://www.epa.gov/oig/reports/2006/20060801-2006-P-00028.pdf.

[r38] U.S. EPA (U.S. Environmental Protection Agency). (2006b). Permethrin Facts (Reregistration Eligibility Decision Fact Sheet).. http://www.epa.gov/oppsrrd1/REDs/factsheets/permethrin_fs.htm.

[r39] Whyatt RM Garfinkel R Hoepner LA Andrews H Holmes D Williams MK 2009 A biomarker validation study of prenatal chlorpyrifos exposure within an inner-city cohort during pregnancy. Environ Health Perspect 117 559 567; 10.1289/ehp.0800041 19440494 PMC2679599

[r40] Willet W. (1998). Nutritional Epidemiology. 2nd ed.. New York:Oxford University Press.

